# Body Weight and Scrotal-Testicular Biometry in Three Indigenous Breeds of Bucks in Arid and Semiarid Agroecologies, Ethiopia

**DOI:** 10.1155/2017/5276106

**Published:** 2017-05-02

**Authors:** Amare Eshetu Gemeda, Kefelegn Workalemahu

**Affiliations:** College of Veterinary Medicine, Haramaya University, P.O. Box 138, Dire Dawa, Ethiopia

## Abstract

The body weight and testicular and epididymal parameters of Afar, Long-eared Somali (LES), and Woyto-Guji (WG) breeds of goat were investigated. A total of 405 randomly selected bucks of Afar (*n* = 135), Long-eared Somali (*n* = 135), and Woyto-Guji (*n* = 135) were included in this study. The overall mean scrotal circumference (SC), testicular volume (TV), testicular length (TL), testicular weight (TW), body weight (BW), epididymal weight (EW), body condition score, and testicular diameter (TD) measurements in all bucks were 20.8 ± 1.94 cm, 68.1 ± 6.18, 4.96 ± 0.79 cm, 70.0 ± 5.66 g, 22.1 ± 2.98 Kg, 9.09 ± 1.88 g, 2.55 ± 0.68, and 4.28 ± 0.45 cm, respectively. Significant (*p* < 0.05) breed differences in SC, TD, TL, TW, BW, EW, and TV were recorded. Long-eared Somali (LES) breed was heaviest and Afar breed was the lightest and Woyto-Guji (WG) had the average BW. In all breeds, the parameters were positively correlated. In Afar breed, the TW had a significant correlation with BW (*r* = 0.90) and SC (*r* = 0.65). In LES BW was highly correlated with TD (*r* = 0.96) and TL (*r* = 0.96). In WG, TW was significantly correlated with TD (*r* = 0.94), EW (*r* = 0.90), TL (*r* = 0.89), and BW (*r* = 0.82). In multiple regression analysis the linear combinations of BCS, SC, and BW significantly predicted TW, TL, TV, TD, and EW in all breeds. In conclusion, Long-eared Somali breed displayed greater BW and scrotal and testicular traits.

## 1. Introduction

In Ethiopia nearly 22.78 million goats are reared in mixed, pastoral, and agropastoral production systems in extensive farming for different purposes such as sociocultural reasons, milk, meat production, and cash generation [1, 2]. These goats due to their small body size, short reproductive cycle, broad feeding habits, and adaptation strategy to unfavorable environmental conditions such as scarce feed resources are preferred by framers [3–6]. Moreover incorporating goats in grazing systems of a mixed animal species results in efficient use of the natural resources with supple management of livestock environments [5].

Reproductive parameters determine several aspects of goat production including genetic improvement, so adequate data on reproduction in native breeds of goats in Ethiopia is essential for reproductive management and to set up feasible breeding schema [7, 8]. In goat's production, buck fertility influences flock performance and reproductive efficiency compared to the fertility of individual doe [7]; thus, selection of highly fertile bucks is vital for improved goat production [9, 10]. Therefore, to improve goat production in the tropics, the reproductive efficiency and fertility of the bucks require attention [11]. In contrast, fertility studies in livestock have generally tended to focus on the female side with less emphasis on the male [11].

Data on goat reproductive organs morphometric values is useful in breeding soundness evaluation (BSE) to determine fertility potential of breeding males. Such data includes scrotal circumference measurements, an integral part of BSE of animals with a pendulous scrotum [12], particularly, in bulls due to their high correlations with testicular size and sperm production capacity [12, 13]. Parameters such as body size and testicular measurements are also commonly employed in breeding soundness evaluations [14]. Among the selection criteria, testis size is the most suitable parameter to indirectly improve the reproductive performance of females [15, 16]. Furthermore, testicular size is a reliable parameter of the status of reproductive growth, spermatogenesis, and seminal characteristics [17]. However, most works of these natures have been done in bulls but it also relates to other species [18]. Also, information on similar relationship is limited in bucks [13].

The Long-eared Somali, Woyto-Guji, and Afar goats are large-, medium-, and small-sized animals, respectively, that dominate a large part of the arid and semiarid agroecologies of Ethiopia. The Afar breed is distributed in the Rift Valley strip, Danakil depression, Gewane, and northern and western Hararghe, whereas the Long-eared Somali breed is distributed in Ogaden, the lowlands of Bale, Borena, and southern Sidama, and the WG breed is distributed in North and South Omo, southern Sidama, and parts of Wolayta. These goat breeds are adapted to arid and semiarid agroclimates and managed mainly under the pastoral farming systems [19–21]. They represent dominant goat breeds of the slaughter flock in the modern export abattoirs in Ethiopia, which export goat meat to the Middle East [7]. In Ethiopia, prior studies on indigenous goat breeds were focused on the reproductive traits and fertility of female not male goats; as a result, data on fertility and male reproductive traits in indigenous goats in Ethiopia is scant [22, 23]. In view of these, parameters that are relevant to breeding soundness of buck should be assessed. Therefore, the present study was designed and conducted to examine body weight and scrotal and testicular parameters in three indigenous goat breeds in arid and semiarid parts of Ethiopia.

## 2. Materials and Methods

### 2.1. Study Area

The study was conducted on apparently health bucks presented for slaughter at Luna Export Abattoir located in Modjo (8°35′N, 39°7′E) town, Lume district, Eastern Shewa Administrative Zone of Oromia Regional State, central Ethiopia. Modjo is located 73 kilometers southeast of Addis Ababa. Luna Export Abattoir exports fresh chilled small ruminant, mainly young male goat meat to the Middle East and African countries [24].

### 2.2. Study Animals and Data Collection

This study was conducted on apparently healthy bucks which belong to three Ethiopian indigenous goat breeds, namely, Afar locally named Adal or Danakil; Woyto-Guji (WG) locally named Woyto, Guji, and Konso; and Long-eared Somali (LES): its local names Degheir, Galla, Digodi, and Melebo [19, 21]. The breeds of the goat were categorized as per the physical characteristics described by FARM-Africa [19] and ESGPIP [21]. A total of 405 bucks including 135 animals in each breed were randomly selected from a population of bucks presented for slaughter. Accordingly, for respective breed study goats were grouped into three age classes, that is, less than 1 year; 1 to 2 years; and more than 2 years of age. Bucks were grouped according to their age using the dentition method for African indigenes goat [25]. Each buck considered for this study was assigned an identifier number that was used for both ante- and postmortem examinations. Prior to slaughter of bucks their testes and scrotum were examined for size, symmetry, and consistence as described by [13]. During antemortem examination genital organs of each animal were cautiously examined for the presence of any abnormalities and bucks with signs of clinical problems and gross abnormalities of reproductive organs were omitted from this study [24]. Thus, animals included in this study were confirmed to be free of any gross ante- or postmortem abnormalities and disorders of the genital organs.

At antemortem study prior to the slaughter, live body parameters such as age, body condition score (BCS), body weight (BW, Kg), and scrotal circumference (SC, cm) of each animal were examined and recorded. Body condition score was evaluated as per the method described by Steele [26], Ford et al. [27], and Okere et al. [28]. The scores ranged from 1 = emaciated to 5 = obese. The live body weight (BW; kg) of each animal was recorded before slaughter using a portable balance. Scrotal circumference (SC) was measured according to the method described by Goyal and Memon [13] using nonstretchable measuring tape.

Immediately after bucks were slaughtered, the testes with epididymides of each animal were collected and put into plastic bags labeled with animals particularities and transported in an ice pack to the laboratory where the epididymides were separated from testes and then testicular and epididymal parameters including testicular weights (TW, g), testicular diameter (TD, cm), testicular length (TL, cm), testicular volume (TV), and epididymal weights (EW, g) were measured and recorded for each animal according to methods and procedures adopted from Oyeyemi et al. [29] and Ajao et al. [11]. In brief, testicular weight (TW) and epididymal weight (EW) in gram (g) were measured using a general purpose weighing balance. Testicular diameter (cm) was measured around the widest point at an area that is equidistant to the testicular poles. Testicular length (cm) was also measured along the longitudinal axis of the testis beginning from one pole of the testis to the other pole, while testicular volume (TV, ml) was measured by using a water displacement technique [11, 29, 30].

### 2.3. Statistical Analysis

The right and left testicular and epididymal measurements were obtained separately; however, the average values of the paired organs were used in the analysis. The data obtained were summarized as mean ± standard deviation and were analyzed using statistical tools of SPSS for Windows version 17.0 (SPSS Inc., Chicago, IL, USA). The effect of breed on body condition (BCS), body weight (BW), and testicular/epididymal measurements was analyzed by one-way analysis of variance (ANOVA). Correlations among the testicular and epididymal parameters with body weight and between the parameters were evaluated. Separate prediction models (multiple regression equations) were developed for each breed to predict testicular and epididymal parameters.

The final model fitted was set out using the following equation [31]:

(1) $$\begin{array}{c} Y_{i}=B_{0}+B_{1}X_{1}+B_{2}X_{2}+B_{3}X_{3}+\cdots +B_{n}X_{n}, \end{array}$$

where *Y*_*i*_ is the testicular weights (TW), testicular diameter (TD), testicular length (TL), testicular volume (TV), and epididymal weights (EW); *X*_1_,…, *X*_*n*_ are the body condition, scrotal circumference, and body weight; *B*_0_ is the intercept; *B*_1_,…, *B*_*n*_ are the multiple regression coefficients of the independent variables *X*_1_,…, *X*_*n*_.

## 3. Results

The mean body weight, scrotal circumference, epididymal weight, and testicular traits with respect to breed are shown in Table 1. The overall scrotal circumference (SC), testicular volume (TV), testicular length (TL), testicular weight (TW), body weight (BW), epididymal weight (EW), body condition score, and testicular diameter (TD) measurements in all bucks were 20.8 ± 1.94 cm, 68.1 ± 6.18, 4.96 ± 0.79 cm, 70.0 ± 5.66 g, 22.1 ± 2.98 Kg, 9.09 ± 1.88 g, 2.55 ± 0.68, and 4.28 ± 0.45 cm, respectively.

Analysis and comparison of measurements in the three indigenous breeds show that Afar, Long-eared Somali (LES), and Woyto-Guji (WG) breeds display significant differences (*p* < 0.05) in scrotal circumference (SC), testicular volume (TV), testicular length (TL), testicular weight (TW), body weight (BW), epididymal weight (EW), and testicular diameter (TD). Long-eared Somali (LES) breed scored significantly higher measurement values for most of the traits followed by Woyto-Guji (WG) and Afar breeds.

Results of the correlation analysis between live body and epididymal and testicular measurements as described by Pearson correlation coefficients in Afar, Long-eared Somali, and Woyto-Guji breeds are summarized in Tables 2 and 3. In Afar bucks the testes weight had a highly significant correlation with body weight (*r* = 0.90, *p* < 0.01), testicular length (*r* = 0.86, *p* < 0.01), testicular diameter (*r* = 0.80, *p* < 0.01), testicular volume (*r* = 0.64, *p* < 0.01), and scrotal circumference (*r* = 0.65, *p* < 0.01). In Long-eared Somali (LES) breed, the highest correlation coefficients were observed for body weight and testicular diameter (*r* = 0.96, *p* < 0.01) and body weight and testicular length (*r* = 0.96, *p* < 0.01), followed by testicular diameter and testicular length (*r* = 0.92, *p* < 0.01) and body weight and testicular weight (*r* = 0.91, *p* < 0.01). In Woyto-Guji (WG) breed, testicular weight was highly and significantly correlated with testicular diameter (*r* = 0.94, *p* < 0.01), epididymal weight (*r* = 0.90, *p* < 0.01), testicular length (*r* = 0.89, *p* < 0.01), and body weight (*r* = 0.82, *p* < 0.01).

The regression coefficients and *R*^2^ values of testicular variables and epididymal weight from live body parameters estimation with their level of significance using linear regression models for Afar breed are shown in Table 4. The linear combinations of body condition, scrotal circumference, and body weight significantly predicted testicular and epididymal traits in Afar bucks (*p* < 0.001). In Afar breed, the standardized beta coefficients suggested that scrotal circumference contributed most to predicting testicular diameter and epididymal weight, whereas body weight stood out as most linear predictor for testicular weight. Testicular length of Afar bucks was best predicted by scrotal circumference and body weight. Testicular volume was best predicted by scrotal circumference, body weight, and body condition. Thus, an Afar bucks, weighing 18 Kg with a BCS of 3 and scrotal circumference of 18 cm, would have testicular weight, testicular length, testicular diameter, epididymal weight, and testicular volume of 64.52 g, 5.14 cm, 3.37 cm, 9.19 g, and 65.15, respectively.

Multivariable regression analysis of testicular and epididymal biometrics on live body parameters in Long-eared Somali bucks was also computed (Table 5). In Long-eared Somali bucks body weight and scrotal circumference were found to be good estimate of testicular weight, length, and diameter and epididymal weight. The predicted testicular weight, testicular length, testicular diameter, epididymal weight, and testicular volume of a Long-eared Somali buck breed weighing 26 kg with a BCS of 4 and scrotal circumference of 24 cm were 80.98 g, 5.71 cm, 4.73 cm, 12.44 g, and 72.94, respectively.

The *R*^2^ values and regression coefficients of testicular and epididymal traits from body parameters estimation with their level of significance based on linear regression models in Woyto-Guji bucks are shown in Table 6. Body weight contributed most to predicting testicular and epididymal weight, testicular diameter, and length, although scrotal circumference stood out as most linear predictor for testicular volume of Woyto-Guji bucks. Therefore, Woyto-Guji buck with body weight of 20 Kg, scrotal circumference of 20 cm, and a BCS of 3 would have 64.81 g, 3.82 cm, 4.02 cm, 6.96 g, and 66.64 of testicular weight, testicular length, testicular diameter, epididymal weight, and testicular volume, respectively.

## 4. Discussion

In this study, live body and testicular and epididymal parameters were evaluated in three goat breeds natives to arid and semiarid agroecologies of Ethiopia. The mean (±standard deviation) values of SC (cm), TW (g), TV, TL (cm), TD (cm), and EW (g) were 20.8 ± 1.94, 70.0 ± 5.66, 68.1 ± 6.18, 4.97 ± 0.79, 4.28 ± 0.45, and 9.09 ± 1.88, respectively. The values of the testicular traits obtained in this study are higher than 17.15 ± 1.14 cm, 52.16 ± 10.29 g, and 4.71 ± 0.52 cm reported for scrotal circumference, testicular weight (g), and testicular length, respectively, in Shale bucks in humid zone of Nigeria [29]. But the mean value of testicular diameter in this study is lower than 10.97 ± 0.90 reported in Shale bucks [29]. The discrepancies in values might be due to breed differences, as it has been reported there exist breed differences in the size of the testicular parameters [13].

A measurement of scrotal circumference is an integral part of breeding soundness evaluation of animals with a pendulous scrotum, particularly in bulls [12, 13]. Furthermore, measurements of the SC are viewed as a cheap, repeatable, and objective way to estimate sperm production [32]. In this study the overall mean value of scrotal circumference in bucks was 20.8 ± 1.94 cm and it was observed that the scrotal circumference of Long-eared Somali (21.4 ± 1.67) breed was significantly (*p* < 0.05) higher than that of Afar (20.5 ± 2.10) and Woyto-Guji (20.6 ± 1.93) breeds, but there had been no significant (*p* > 0.05) disparity between Afar and Woyto-Guji breeds. Raji et al. [33] reported SC of 23.99 ± 0.17 and 20.75 ± 0.25 cm in Red Sokoto and Borno White bucks, respectively, in Nigeria which are similar to the results in this study. Furthermore, the mean values of the scrotal circumference in three breeds in the present study are higher than 15.73 cm and 17.15 ± 1.14 cm reported for African Dwarf bucks [34] and Shale bucks [29], respectively, but it was lower than 28 to 39 cm in Nubian buck [13]. These differences might be due to the effect of genotype or breed; similar reports of differences among breeds have been reported in goats [33]. Furthermore, studies suggest that, in addition to breed, season, nutrition, and body weight had an effect on SC of domestic animals [35, 36]. Thus, in this study difference in nutritional managements of bucks could also be responsible for the differences in scrotal circumference among breeds. The larger scrotal circumference value for Long-eared Somali breed, as compared to Afar and Woyto-Guji breeds, might be due to differences in size, as large-sized breeds have greater SC [20, 37]. SC is reliable measurement of reproductive status and seminal characteristics in goats [17]. For instance, a decrease in SC is an indication of an increase in morphologically abnormal sperm in an ejaculate [38]. Hence, Söderquist and Hultén [32] recommended that SC measurements could be applied to estimate testes weight and fertility in rams.

Body weight and condition influence the reproductive potential of domestic animals and body weight itself is affected by breed, age, and nutritional status of bucks [36]. In Ethiopia, Long-eared Somali, Woyto-Guji, and Afar breeds of goats are considered as large-, medium-, and small-sized animals, respectively [19, 21]. In this study the body weight was significantly (*p* < 0.05) varied between breeds. For Long-eared Somali, the mean body weight was 24.5 ± 2.71 kg, Woyto-Guji had intermediate weight of 21.9 ± 1.84 kg, and Afar breed recorded the lightest weight of 19.7 ± 2.16 kg. Breeding soundness examination of male domestic animals should consider body condition because spermatogenesis tends to be limited in poor body condition; thus, sires should be maintained in moderate condition [39]. In this study there were no significant breed (*p* > 0.05) variations in body condition score. However, Long-eared Somali, Woyto-Guji, and Afar breeds had higher body condition score than Arsi-Bale and Central highlands breeds of bucks [20]. The differences might be due to breed difference, nutritional status, and variations in climatic conditions.

Combined with other variables testicular weight can be used to select males for testicular size at puberty since it is a reliable variable for estimating the sperm production capacity [40] but it varies depending on the breed [41]. In this study the Long-eared Somali breed had a significantly (*p* < 0.05) heavier testes weight than the Woyto-Guji and Afar breeds. The values recorded in the current study are lower than 98.4 ± 1.36 and 92.1 ± 1.88 reported in Red Sokoto and Borno White breeds in a semiarid region of Nigeria [33] but higher than 52.16 ± 10.29 reported for Shale bucks [29]. These differences might be due to differences in breed and nutritional management [41]. It has been reported that animals with heavier testes produce more spermatozoa than those with smaller testes [42]. Furthermore, the mean testes volume of the Long-eared Somali breed was significantly (*p* < 0.05) higher than that of the Woyto-Guji and Afar breeds. These disparities in the mean testes volume for the different breeds recorded in the current study agree with the report of [41] in different breeds of goats in Nigeria. In this study significant (*p* < 0.05) breed difference on epididymal weight, testicular diameter, and testicular length was apparent where Long-eared Somali breed scored significantly higher values for testicular diameter and testicular length followed by Woyto-Guji and the Afar breeds had the least values. On the other hand, epididymal weight was highest in Long-eared Somali breed followed by Afar breed and Woyto-Guji breed scored the least value.

In domestic animals, testes size and sperm production are highly correlated which implies that the larger the testes, the greater the sperm production [18]. Also, SC is good predictor of testicular size along with strong and positive associations between them [18]. In this study, in LES breed, SC had strongest correlations with TW (*r* = 0.88, *p* < 0.01) and body weight (*r* = 0.86, *p* < 0.01), while in Afar breed correlations of SC and TW (*r* = 0.65, *p* < 0.01) and SC and BW (*r* = 0.54, *p* < 0.01) were not that strong compared to that of LES. Furthermore, the correlations of SC with TW (*r* = 0.77, *p* < 0.01) and SC with that of BW (*r* = 0.66, *p* < 0.01) in WG breed were moderate. Raji et al. [33] reported the correlation coefficient of 0.81 between SC and BW in Borno white goats in Nigeria which is similar to that recorded for LES, but higher than values reported for Afar and WG breeds in this study. Furthermore, the correlation coefficients between BW and SC for LES (*r* = 0.86) and WG (*r* = 0.66) are higher than (*r* = 0.53) the correlation reported for Ogaden bucks in Ethiopia [36] which is similar to (*r* = 0.54) correlation recorded for Afar bucks in this study. They were however lower than (*r* = 0.94) correlation reported for Saneen and Jamnapuri crosses [43]. The correlation coefficients of 0.88 and 0.77 between SC and TW for LES and WG breeds, respectively, were comparable to (*r* = 0.79) that reported for red Sokoto bucks [33], which was higher than that (*r* = 0.65) recorded in Afar bucks in this study. But the values obtained in this study were lower than that (*r* = 0.96) reported in Borno white goats [33]. These differences might be due to the effect of genotype or breed [33].

BW had high and significant correlation with TW in LES (*r* = 0.91, *p* < 0.001), Afar (*r* = 0.90, *p* < 0.001), and WG (*r* = 0.82, *p* < 0.001) breeds indicating a good association between BW and TW. Similar reports of BW being significantly correlated with testicular weight were observed in different breeds of goats [11, 44]. These high, positive, and significant correlations between body weight and SC with testicular measurements suggest that either of these variables or their combinations could provide a good estimate for predicting testicular and epididymal traits. Testes weight is known to be very highly correlated with testicular sperm reserves [45]; thus males with larger testes tend to produce more sperm [46]. It has been reported that measurement of scrotal circumference, testes length, weight, and width would be a reliable predictor of the sperm producing capacity of bucks and they can be used to select for improved sperm production and breeding males [47]. In multiple linear regression analysis, simultaneous addition of body condition, scrotal circumference, and body weight were found to increase reliability of predicting testicular parameters in all three breeds. Body weight and SC were found to be a significant and good estimate of testicular measurements and EW with large standardized beta in Afar, Long-eared Somali, and Woyto-Guji breeds.

In conclusion, this study highlighted body weight and reproductive traits in bucks of three breeds native to arid and semiarid agroecologies of Ethiopia. Breed was found to influence body weight, SC, epididymal weight, and testicular biometry in Afar, Long-eared Somali, and Woyto-Guji breeds. Long-eared Somali breed displayed higher body weight, SC, and testicular measurements. The most suitable live body measurements that can be easily measured on live bucks and could be used to predict epididymal and testicular biometry were found to be SC and BW. The findings of this study could be used in breeding soundness assessment to select fertile bucks in their natural environments.

## Figures and Tables

**Table 1:** Mean body weight, body condition, and scrotal and testicular measurements in bucks of three breeds in arid and semiarid agroecologies, Ethiopia (mean ± SD).

Measurements	Breeds			
	Afar (*n* = 135)	LES (*n* = 135)	WG (*n* = 135)	Overall
Scrotal circumference	20.5 ± 2.10^a^	21.4 ± 1.67^b^	20.6 ± 1.93^a^	20.8 ± 1.94
Testicular volume	67.1 ± 6.77^a^	69.8 ± 5.83^b^	67.3 ± 5.92^a^	68.1 ± 6.18
Testicular length	4.47 ± 0.90^a^	5.44 ± 0.55^b^	4.99 ± 0.57^c^	4.97 ± 0.79
Testicular weight	67.6 ± 3.49^a^	74.8 ± 5.81^b^	67.6 ± 3.97^a^	70.0 ± 5.66
Body weight	19.7 ± 2.16^a^	24.5 ± 2.71^b^	21.9 ± 1.84^c^	22.1 ± 2.98
Epididymal weight	10.7 ± 1.20^a^	11.1 ± 1.44^b^	7.92 ± 1.06^c^	9.09 ± 1.88
Testicular diameter	4.06 ± 0.24^a^	4.59 ± 0.49^b^	4.19 ± 0.39^c^	4.28 ± 0.45
Body condition score	2.52 ± 0.71^a^	2.59 ± 0.50^a^	2.54 ± 0.71^a^	2.55 ± 0.68

^a,b,c^Means in the same row and with different superscript have significant difference (*p* < 0.05). LES: Long-eared Somali; WG: Woyto-Guji.

**Table 2:** Correlations between live weight and testicular/epididymal traits in Afar and Long-eared Somali (LES) breeds.

	BCS	SC	TV	TL	TD	TW	BW	EW
BCS	1	0.13^ns^	0.29^*∗∗*^	0.18^*∗*^	0.26^*∗∗*^	0.28^*∗∗*^	0.17^ns^	0.19^*∗*^
SC	0.42^*∗∗*^	1	0.44^*∗∗*^	0.82^*∗∗*^	0.83^*∗∗*^	0.65^*∗∗*^	0.54^*∗∗*^	0.87^*∗∗*^
TV	0.27^*∗∗*^	0.32^*∗∗*^	1	0.61^*∗∗*^	0.60^*∗∗*^	0.64^*∗∗*^	0.60^*∗∗*^	0.43^*∗∗*^
TL	0.48^*∗∗*^	0.83^*∗∗*^	0.22^*∗*^	1	0.97^*∗∗*^	0.86^*∗∗*^	0.82^*∗∗*^	0.73^*∗∗*^
TD	0.38^*∗∗*^	0.85^*∗∗*^	0.27^*∗∗*^	0.92^*∗∗*^	1	0.80^*∗∗*^	0.78^*∗*^	0.80^*∗∗*^
TW	0.49^*∗∗*^	0.88^*∗∗*^	0.17^*∗*^	0.90^*∗∗*^	0.88^*∗∗*^	1	0.90^*∗∗*^	0.52^*∗∗*^
BW	0.41^*∗∗*^	0.86^*∗∗*^	0.23^*∗∗*^	0.96^*∗∗*^	0.96^*∗∗*^	0.91^*∗∗*^	1	0.47^*∗∗*^
EW	0.36^*∗∗*^	0.76^*∗∗*^	0.10^ns^	0.82^*∗∗*^	0.68^*∗∗*^	0.80^*∗∗*^	0.82^*∗∗*^	1

BSC: body condition score; SC: scrotal circumference; TV: testicular volume; TL: testicular length; TW: testicular weight; BW: body weight; EW: epididymal weight; TD: testicular diameter; ^*∗∗*^*p* < 0.01; ^*∗*^*p* < 0.05; ns: not significant. Values above the diagonal are for Afar while those below are for Long-eared Somali goats.

**Table 3:** Correlations between live body measurements and epididymal/testicular traits in Woyto-Guji breed.

	BCS	SC	TV	TL	TD	TW	BW	EW
BCS	1	0.15^ns^	0.24^*∗∗*^	0.36^*∗∗*^	0.40^*∗∗*^	0.40^*∗∗*^	0.37^*∗∗*^	0.40^*∗∗*^
SC	0.15^ns^	1	0.67^*∗∗*^	0.67^*∗∗*^	0.72^*∗∗*^	0.77^*∗∗*^	0.66^*∗∗*^	0.66^*∗∗*^
TV	0.24^*∗∗*^	0.67^*∗∗*^	1	0.38^*∗∗*^	0.51^*∗∗*^	0.55^*∗∗*^	0.41^*∗∗*^	0.36^*∗∗*^
TL	0.36^*∗∗*^	0.67^*∗∗*^	0.38^*∗∗*^	1	0.96^*∗∗*^	0.89^*∗∗*^	0.95^*∗∗*^	0.98^*∗∗*^
TD	0.40^*∗∗*^	0.72^*∗∗*^	0.51^*∗∗*^	0.96^*∗∗*^	1	0.94^*∗∗*^	0.92^*∗∗*^	0.96^*∗∗*^
TW	0.40^*∗∗*^	0.77^*∗∗*^	0.55^*∗∗*^	0.89^*∗∗*^	0.94^*∗∗*^	1	0.82^*∗∗*^	0.90^*∗∗*^
BW	0.37^*∗∗*^	0.66^*∗∗*^	0.41^*∗∗*^	0.95^*∗∗*^	0.92^*∗∗*^	0.82^*∗∗*^	1	0.94^*∗∗*^
EW	0.40^*∗∗*^	0.66^*∗∗*^	0.36^*∗∗*^	0.98^*∗∗*^	0.96^*∗∗*^	0.90^*∗∗*^	0.94^*∗∗*^	1

BSC: body condition score; SC: scrotal circumference; TV: testicular volume; TL: testicular length; TW: testicular weight; BW: body weight; EW: epididymal weight; TD: testicular diameter; ^*∗∗*^*p* < 0.01; ns: not significant.

**Table 4:** Multivariable regression analysis of testicular and epididymal parameters of Afar bucks in Ethiopia.

Parameter	Predictors	*B*	S. error	Beta	*t*	Sig.	*R*	*R* ^2^	Adjusted *R*^2^
TW	Constant	33.8	1.27		26.6	*∗∗∗*	0.93	0.87	0.86
	SC	0.38	0.07	0.22	5.79	*∗∗∗*			
	BW	1.23	0.06	0.77	20.1	*∗∗∗*			
	BCS	0.58	0.16	0.12	3.68	*∗∗∗*			

TL	Constant	−4.07	0.31		−12.9	*∗∗∗*	0.94	0.88	0.87
	SC	0.24	0.02	0.53	14.7	*∗∗∗*			
	BW	0.22	0.02	0.53	14.7	*∗∗∗*			
	BCS	0.31	0.34	0.02	0.79	ns			

TD	Constant	1.83	0.08		22.1	*∗∗∗*	0.93	0.86	0.85
	SC	0.06	0.004	0.56	14.4	*∗∗∗*			
	BW	0.05	0.004	0.46	11.6	*∗∗∗*			
	BCS	0.03	0.01	0.12	3.43	*∗∗*			

EW	Constant	−0.5	0.58		−0.86	ns	0.87	0.77	0.76
	SC	0.52	0.03	0.87	17.2	*∗∗∗*			
	BW	−0.01	0.03	−0.02	−0.29	ns			
	BCS	0.14	0.07	0.08	1.93	ns			

TV	Constant	22.4	5.18		4.33	*∗∗∗*	0.65	0.42	0.41
	SC	0.54	0.27	0.16	1.99	*∗*			
	BW	1.53	0.25	0.49	6.10	*∗∗∗*			
	BCS	1.83	0.65	0.19	2.84	*∗∗*			

BSC: body condition score, SC: scrotal circumference, TV: testicular volume, TL: testicular length, TW: testicular weight, BW: body weight, EW: epididymal weight, and TD: testicular diameter. ^*∗∗∗*^*p* < 0.001, ^*∗∗*^*p* < 0.01, ^*∗*^*p* < 0.05, and ns: not significant.

**Table 5:** Multivariable regression analysis of testicular and epididymal parameters of Long-eared Somali bucks in Ethiopia.

Parameter	Predictors	*B*	S. error	Beta	*t*	Sig.	*R*	*R* ^2^	Adjusted *R*^2^
TW	Constant	22.4	1.87		11.9	*∗∗∗*	0.93	0.87	0.87
	SC	1.00	0.17	0.36	5.82	*∗∗∗*			
	BW	1.19	0.13	0.55	9.02	*∗∗∗*			
	BCS	0.91	0.28	0.11	3.21	*∗∗*			

TL	Constant	0.21	0.14		1.55	ns	0.96	0.92	0.92
	SC	0.01	0.01	0.03	0.06	ns			
	BW	0.19	0.01	0.91	19.2	*∗∗∗*			
	BCS	0.08	0.02	0.11	4.02	*∗∗∗*			

TD	Constant	0.17	0.12		1.46	ns	0.96	0.93	0.93
	SC	0.02	0.01	0.90	1.93	ns			
	BW	0.16	0.01	0.89	19.4	*∗∗∗*			
	BCS	−0.02	0.02	−0.03	−1.09	ns			

EW	Constant	−0.22	0.73		−0.30	ns	0.82	0.68	0.67
	SC	0.15	0.07	0.22	2.21	*∗*			
	BW	0.33	0.05	0.63	6.49	*∗∗∗*			
	BCS	0.12	0.11	0.01	0.13	ns			

TV	Constant	50.0	4.87		10.3	*∗∗∗*	0.36	0.13	0.13
	SC	1.14	0.45	0.41	2.54	*∗*			
	BW	−0.41	0.34	−0.19	−1.19	ns			
	BCS	1.56	0.74	1.97	1.97	ns			

BSC: body condition score, SC: scrotal circumference, TV: testicular volume, TL: testicular length, TW: testicular weight, BW: body weight, EW: epididymal weight, and TD: testicular diameter. ^*∗∗∗*^*p* < 0.001, ^*∗∗*^*p* < 0.01, ^*∗*^*p* < 0.05, and ns: not significant.

**Table 6:** Multivariable regression analysis of testicular and epididymal parameters of Woyito-Guji bucks in Ethiopia.

Parameter	Predictors	*B*	S. error	Beta	*t*	Sig.	*R*	*R* ^2^	Adjusted *R*^2^
TW	Constant	22.97	2.05		11.24	*∗∗∗*	0.89	0.79	0.78
	SC	0.92	0.11	0.44	8.14	*∗∗∗*			
	BW	1.01	0.13	0.47	8.09	*∗∗∗*			
	BCS	1.08	0.29	0.16	3.74	*∗∗∗*			

TL	Constant	−2.21	0.19		−11.4	*∗∗∗*	0.95	0.91	0.91
	SC	0.02	0.01	0.07	1.96	ns			
	BW	0.28	0.01	0.90	23.9	*∗∗∗*			
	BCS	0.01	0.03	0.01	0.49	ns			

TD	Constant	0.64	0.12		5.34	*∗∗∗*	0.93	0.87	0.87
	SC	0.04	0.01	0.23	5.43	*∗∗∗*			
	BW	0.12	0.01	0.72	16.4	*∗∗∗*			
	BCS	0.06	0.02	0.12	3.57	*∗∗*			

EW	Constant	−4.20	0.39		−10.6	*∗∗∗*	0.94	0.89	0.89
	SC	0.05	0.02	0.08	2.08	*∗*			
	BW	0.49	0.02	0.86	20.4	*∗∗∗*			
	BCS	0.12	0.06	0.07	2.18	*∗*			

TV	Constant	24.95	4.80		5.19	*∗∗∗*	0.69	0.47	0.46
	SC	2.29	0.27	0.74	8.61	*∗∗∗*			
	BW	−0.48	0.29	−0.15	−1.64	ns			
	BCS	1.83	0.68	0.19	2.72	*∗∗*			

BSC: body condition score, SC: scrotal circumference, TV: testicular volume, TL: testicular length, TW: testicular weight, BW: body weight, EW: epididymal weight, and TD: testicular diameter. ^*∗∗∗*^*p* < 0.001, ^*∗∗*^*p* < 0.01, ^*∗*^*p* < 0.05, and ns: not significant.

## References

[1] Tesfaye A. (2004). *Genetic Characterization of Indigenous Goat Populations of Ethiopia Using Microsatellite DNA Markers [Ph.D. thesis]*.

[2] CSA (Central Statistical Authority) (2013). *Agricultural Sample Survey Volume II: Report on Livestock and Livestock Characteristics in Ethiopia*.

[3] Misra A. K., Singh K. (2002). Effect of water deprivation on dry matter intake, nutrient utilization and metabolic water production in goats under semi-arid zone of India. *Small Ruminant Research*.

[4] IBC (Institute of Biodiversity Conservation) (2004). *The State of Ethiopia’s Farm Animal Genetic Resources: Country Report. A Contribution to the First Report on the State of the World’s Animal Genetic Resources*.

[5] Hirpa A., Abebe G., Yami and A., Merkel R. C. (2008). Economic significance of sheep and goat. *Sheep and Goat Production Handbook for Ethiopia*.

[6] Umeta G., Duguma M., Hundesa F., Muleta M. (2011). Participatory analysis of problems limiting goat production at selected districts of East Showa zone, Ethiopia. *African Journal of Agricultural Research*.

[7] Yoseph M. G. (2007). *Reproductive Traits in Ethiopian Male Goats with Special Reference to Breed and Nutrition [Ph.D. thesis]*.

[8] Alemu A. T. (2007). *Phenotypic Characterization of Indigenous Goat Types and Their Production System in Shabelle Zone, South Eastern Ethiopia [M. S. thesis]*.

[9] Chacón J., Pérez E., Müller E., Söderquist L., Rodríguez-Martínez H. (1999). Breeding soundness evaluation of extensively managed bulls in Costa Rica. *Theriogenology*.

[10] Memon M. A., Mickelsen W. D., Goyal H. O., Youngquist R. S., Threlfall W. R. (2007). Examination of the reproductive tract and evaluation of potential breeding soundness in bucks. *Current Therapy in Large Animal Theriogenology 2*.

[11] Ajao E. O., Akinyemi M. O., Ewuola E. O., Osaiyuwu O. H. (2014). Body measurements of red Sokoto bucks in Nigeria and their relationship with testicular biometrics. *Iranian Journal of Applied Animal Science*.

[12] Jainudeen M. R., Wahid H., Hafez E. S. E., Hafez E. S. E., Hafez B. (1993). Sheep and goats. *Reproduction in Farm Animals*.

[13] Goyal H. O., Memon M. A., Youngquist R. S., Threlfall W. R. (2007). Clinical reproductive anatomy and physiology of the buck. *Current Therapy in Large Animal Theriogenology*.

[14] Agga G. E., Udala U., Regassa F., Wudie A. (2011). Body measurements of bucks of three goat breeds in Ethiopia and their correlation to breed, age and testicular measurements. *Small Ruminant Research*.

[15] Walkley J. R. W., Smith C. (1980). The use of physiological traits in genetic selection for litter size in sheep. *Journal of Reproduction and Fertility*.

[16] Schoeman S. J., Els H. C., Combrink G. C. (1987). A preliminary investigation into the use of testis size in cross-bred rams as a selection index for ovulation rate in female relatives. *South African Journal of Animal Sciences*.

[17] Daudu C. S. (1984). Spermatozoa output, testicular sperm reserve and epididymal storage capacity of the Red Sokoto goats indigenous to northern Nigeria. *Theriogenology*.

[18] Bearden H. J., Fuquay J. W., Willard S. T. (2004). *Applied Animal Reproduction*.

[19] FARM-Africa Goat Types of Ethiopia and Eritrea. Physical Description and Management Systems.

[20] Mekasha Y., Tegegne A., Abera A., Rodriguez-Martinez H. (2008). Body size and testicular traits of tropically-adapted bucks raised under extensive husbandry in Ethiopia. *Reproduction in Domestic Animals*.

[21] ESGPIP (2009). Ethiopia Sheep and Goat Productivity Improvement Program, Goat Breeds of Ethiopia: a Guide for Identification And Utilization. *Technical Bulletin*.

[22] Solomon A., Workalemahu A., Jabbar M. A., Ahmed M. M., Hurissa B. (2003). Livestock marketing in Ethiopia: a review of structure, performance and development initiatives. *Socio-economics and Policy Research, Working Paper 52*.

[23] Lorato Y., Ahmed K. M., Belay B. (2015). Participatory Ccharacterization of the Woyto-Guji goat and its production environment around Northern Omo, Ethiopia. *Journal of Agriculture and Natural Resources Sciences*.

[24] Dachasa G., Eshetu A. G. (2015). Gross reproductive organs abnormalities in rams and bucks slaughtered at luna export abattoir, Eastern Shewa Zone, Ethiopia. *Journal of Reproduction and Infertility*.

[25] Wilson R. T., Durkin J. W. (1984). Age at permanent incisor eruption in indigenous goats and sheep in semi-arid Africa. *Livestock Production Science*.

[26] Steele M. (1996). *The Tropical Agriculturalist (Goats)*.

[27] Ford J. D., Okere C., Bolden-Tiller O. (2009). Libido test scores, body conformation and traits in Boer and Kiko goat bucks. *ARPN Journal of Agricultural and Biological Sciences*.

[28] Okere C., Bradley P., Bridges E. R., Bolden-Tiller O., Ford D., Paden A. (2011). Relationships among body conformation, testicular traits and semen output in electro-ejaculate pubertal Kiko goat bucks. *ARPN Journal of Agricultural and Biological Sciences*.

[29] Oyeyemi M. O., Fayomi A. P., Adeniji D. A., Ojo K. M. (2012). Testicular and epididymal parameters of Sahel buck in the humid zone of Nigeria. *International Journal of Morphology*.

[30] Toe F., Rege J. E. O., Mukasa-Mugerwa E. (2000). Reproductive characteristics of Ethiopian highland sheep I. Genetic parameters of testicular measurements in ram lambs and relationship with age at puberty in ewe lambs. *Small Ruminant Research*.

[31] Leech N. L., Barrett K. C., Morgan G. A. (2005). *SPSS for Intermediate Statistics: Use and Interpretation*.

[32] Söderquist L., Hultén F. (2006). Normal values for the scrotal circumference in rams of Gotlandic breed. *Reproduction in Domestic Animals*.

[33] Raji A. O., Igwebuike J. U., Aliyu J. (2008). Testicular biometry and its relationship with body weight of indigenous goats in a semi-Arid region of Nigeria. *ARPN Journal of Agricultural and Biological Science*.

[34] Abu A. H., Okwori I. A., Ahemen T., Ojabo L. D. (2016). Evaluation of scrotal and testicular characteristics of west african dwarf bucks fed guava leaf meal. *Journal of Animal Science Advances*.

[35] Mickelsen W. D., Paisley L. G., Dahmen J. J. (1981). The effect of season on the scrotal circumference and sperm motility and morphology in rams. *Theriogenology*.

[36] Mekasha Y., Tegegne A., Rodriguez-Martinez H. (2007). Sperm morphological attributes in indigenous male goats raised under extensive husbandry in Ethiopia. *Animal Reproduction*.

[37] Al-Ghalban A. M., Tabbaa M. J., Kridli R. T. (2004). Factors affecting semen characteristics and scrotal circumference in Damascus bucks. *Small Ruminant Research*.

[38] Mickelsen W. D., Paisley L. G., Dahmen J. J. (1982). Seasonal variations in scrotal circumference, sperm quality, and sexual ability in rams. *Journal of the American Veterinary Medical Association*.

[39] Parkinson T., Noakes D. E., Parkinson T. J., England G. C. W. (2009). Fertility, subfertility and infertility in male animals. *Veterinary Reproduction and Obstetrics*.

[40] Coulter G. H., Larson L. L., Foote R. H. (1975). Effect of age on testicular growth and consistency of Holstein and Angus bulls. *Journal of Animal Science*.

[41] Ibrahim A. A., Aliyu J., Ashiru R. M., Jamilu M. (2012). Biometric study of the reproductive organs of three breeds of sheep in Nigeria. *International Journal of Morphology*.

[42] Brito L. F. C., Silva A. E. D. F., Unanian M. M., Dode M. A. N., Barbosa R. T., Kastelic J. P. (2004). Sexual development in early- and late-maturing Bos indicus and Bos indicus x Bos taurus crossbred bulls in Brazil. *Theriogenology*.

[43] Bongso T. A., Jainudeen M. R., Zahrah A. S. (1982). Relationship of scrotal circumference to age, body weight and onset of spermatogenesis in goats. *Theriogenology*.

[44] Abba Y., Igbokwe I. O. (2015). Testicular and related size evaluations in nigerian sahel goats with optimal cauda epididymal sperm reserve. *Veterinary Medicine International*.

[45] Ogwuegbu S. O., Oko B. O., Akusu M. O., Arie T. A. (1985). Gonadal and extra gonadal sperm reserves of the Maradi (Red Sokoto) goats. *Bulletin of Animal Health and Production in African*.

[46] Okwun O. E., Igboeli G., Ford J. J., Lunstra D. D., Johnson L. (1996). Number and function of Sertoli cells, number and yield of spermatogonia, and daily sperm production in three breeds of boar. *Journal of Reproduction and Fertility*.

[47] Keith L., Okere C., Solaimam S., Tiller O. (2009). Accuracy of predicting body weights from body conformation and testicular morphometry in pubertal Boer goats. *Research Journal of Animal Sciences*.

